# Cyclic AMP: A Polyhedral Signalling Molecule in Plants

**DOI:** 10.3390/ijms21144862

**Published:** 2020-07-09

**Authors:** Emanuela Blanco, Stefania Fortunato, Luigi Viggiano, Maria Concetta de Pinto

**Affiliations:** 1 Institute of Biosciences and Bioresources, National Research Council, Via G. Amendola 165/A, 70126 Bari, Italy; 2 Department of Biology, University of Bari Aldo Moro, Via E. Orabona 4, 70125 Bari, Italy; stefania.fortunato@uniba.it (S.F.); luigi.viggiano@uniba.it (L.V.)

**Keywords:** abiotic stress, cAMP, cyclic nucleotides-gated channels, plant innate immunity

## Abstract

The cyclic nucleotide cAMP (3′,5′-cyclic adenosine monophosphate) is nowadays recognised as an important signalling molecule in plants, involved in many molecular processes, including sensing and response to biotic and abiotic environmental stresses. The validation of a functional cAMP-dependent signalling system in higher plants has spurred a great scientific interest on the polyhedral role of cAMP, as it actively participates in plant adaptation to external stimuli, in addition to the regulation of physiological processes. The complex architecture of cAMP-dependent pathways is far from being fully understood, because the actors of these pathways and their downstream target proteins remain largely unidentified. Recently, a genetic strategy was effectively used to lower cAMP cytosolic levels and hence shed light on the consequences of cAMP deficiency in plant cells. This review aims to provide an integrated overview of the current state of knowledge on cAMP’s role in plant growth and response to environmental stress. Current knowledge of the molecular components and the mechanisms of cAMP signalling events is summarised.

## 1. Introduction

The role of 3′,5′-cyclic adenosine monophosphate (cAMP) as second messenger in a wide variety of physiologic responses has long been unravelled in animals, bacteria, fungi and algae. By contrast, comprehensive knowledge of cAMP signal transduction in higher plants is still lacking. However, over the last twenty years, several pieces of evidence about cAMP biological functions in plants have been reported. Recent advances in plant biology research, supported by biochemical, genetic and omic studies, have led to the characterisation of cAMP as a polyhedral molecule, critically involved in the signalling pathways of both plant development and environmental stress response.

The recognition of cAMP existence in mammals was the first step towards the identification of its role in living organisms [1]. Thereafter, the molecular structure and conformation of cAMP, which are key factors defining cAMP chemical properties, the specificity of target recognition sites and hence its biological activity, have been determined [2,3]. cAMP responses are extremely complex: different stimuli able to change cAMP levels might lead to different physiological outcomes [4]. The high level of cell compartmentalisation of cAMP signalling pathways is the physiological basis of such numerous and diversified responses to cAMP, as signal response elements are differentially localised and temporally regulated [5]. In animals, several interconnected signalling pathways encompass cAMP and cyclic nucleotides activity in the regulation of cellular events, such as cell proliferation, differentiation, death and migration, as well as complex functions, e.g., memory [6,7].

Even though earlier comparative studies put forward a similar role for cAMP in plants, compared to mammalian organisms, cAMP presence and activity in plants has been a matter of controversy for decades. In fact, in plant cells, cAMP is present in nanomolar concentrations, which are one order of magnitude lower than in mammalian cells. In early studies, cAMP was hardly detectable because cellular levels were below the detection limits of available analytical methods [8,9]. The conclusive proof of cAMP existence and activity in plant extracts could be achieved later, through the advances in high performance liquid chromatography and electrospray mass spectrometry, with a lower detection limit of 25 femtomoles for cyclic nucleotide quantification [10,11]. 

More recently, studies in plant cells focused on the biosynthetic molecular components of cAMP production and breakdown, which are able to switch on and off the signal encoded by cAMP. The lifecycle of the cAMP molecule includes a source, several regulatory factors with specific cAMP binding domains to transduce the signal and breakdown enzymes to avoid the accumulation of cAMP and terminate the signal [4]. The identification of plant biosynthetic enzymes was not straightforward and took a lot of efforts because of the low homology with previously characterised animal systems were not straightforward. In animals, cAMP is produced in the cytoplasm from adenosine triphosphate by plasma-membrane associated or soluble adenylate cyclases (ACs). Once generated inside the cell, cAMP transduces signals acting through a few cellular effectors, which are responsible for the divergence of cAMP signalling. Changes in intracellular cAMP levels affect cAMP-dependent protein kinases activity (PKA) [12]. Furthermore, cAMP binds to cyclic nucleotide binding proteins, as cyclic nucleotides-gated channels (CNGCs) and hyperpolarisation-activated cyclic nucleotides-modulated channels [13], or to specific transporters and transcription factors in the nucleus. The cAMP levels are regulated, in terms of both lifetime and cell sub-localisation, by cytoplasmic phosphodiesterases (PDEs), which hydrolyse it into AMP, switching off the signal. 

After the discovery of the first plant AC in *Agapanthus umbellatus* [14], it took considerable efforts before other plant ACs were identified and characterised [15,16,17,18]. Similar to plant guanylate cyclases, also indicated as “moonlighting” proteins [19], which are multifunctional enzymes and hold diverse domain structures, plant ACs also harbour multiple catalytically active AC centres, which co-function with other functional domains [18,20,21,22].

Although physiological and biochemical studies provided evidence for enzyme activation by cyclic nucleotides [23], the lack of genetic information on their molecular identity has hitherto prevented the characterisation of PDEs and PKAs orthologs in plants. However, both PDEs and PKAs are postulated to form complexes with other enzymes [9,23]. There is only one molecularly confirmed PDE in liverwort *Marchantia polymorpha*, which exhibits both AC and PDE activities, but no homologues were found in other plant species [24]. Moreover, many studies point to light PKA activity in many plant species, but these observations still await molecular confirmation [23].

Cyclic nucleotides have a direct effect on cation fluxes (K^+^, Na^+^ and Ca^2+^), and CNGCs are key components of cAMP signal transduction pathways [25]. These ion channels take part to plant reproductive processes, leaf senescence and plant responses to abiotic and biotic stresses [26,27,28,29,30]. They are sensitive to intracellular alterations of the cAMP level and can turn cAMP variations into changes in membrane potential and ion concentrations. CNGCs have different cellular localisation, thus defining the spatial regulation of intracellular cAMP levels.

These findings on plant cAMP biosynthesis and regulation shed light on cAMP role and cAMP-dependent signal transduction mechanisms in plants. However, the understanding of cellular function requires an integrated analysis of context-specific, spatiotemporal data from diverse sources. In this context, the availability of more reliable methods to monitor and/or alter intracellular cAMP levels, without interfering with cell physiological processes, is of utmost importance. Indeed, the roles of cAMP in plants have been mainly established by studies that utilise pharmacological approaches. A recently developed non-invasive method to alter cellular cAMP levels overcame the concern about the effects exerted by the high non-physiological concentrations of exogenously applied cAMPs analogues in both animals and plant systems [31,32,33].

Ion homeostasis [34,35,36], cell division [37,38], pollen tube growth and reorientation [14] and stomatal opening [39,40] are all plant processes involving cAMP level alterations. Proteomic analyses on Arabidopsis plants highlighted the involvement of cAMP in the regulation of photosynthesis and photorespiration, as well as in the energy-transducing pathways and ATP generation [41,42,43]. These studies, while unravelling cAMP role in plant cell development and growth, also pointed out the unavoidable influence of environment in plant life, emphasising cAMP involvement in perception of abiotic and biotic stimuli and in boosting plant stress responses.

In this review, we offer a comprehensive portrayal of molecular mechanisms behind cAMP-dependent signalling events in plant growth and in plant response to abiotic and biotic stress, taking advantage of advanced analytical tools and the newly developed methods successfully applied in plants.

## 2. The cAMP-Sponge, a New Genetic Tool to Unravel cAMP Functions in Plants

Since the assessment of cAMP presence in plants, pharmacological approaches were used to elicit cAMP level alteration inside the cell and to observe associated metabolism changes. Various cell-permeable cAMP analogues and known mammalian activators or inhibitors of ACs or PDEs were initially used to explore cAMP role first in animals and then in plants.

At first, tissues or whole plants were incubated with cAMP for hours or even days, to discover the effects of cAMP on developmental processes, but there was no monitoring of the effective cAMP intake and/or consequent cAMP degradation during the long time of incubation. In plants, the pharmacological approach has been initially used to study cAMP involvement in different physiological processes, spanning from the synthesis of phytoalexins to the control of cell cycle progression [37,38,44]. Alongside, whole-cell patch-clamp assays were performed, where regulators could be directly introduced into the cell or added to the solution, as done for the first time in *Vicia faba*, to investigate cAMP influence on K^+^ channel activities [45]. In addition to these early studies, many other works, even recently, rely on pharmacological methodologies with exogenously applied compounds to alter endogenous cAMP, emphasising cAMP involvement in different plant processes, response to environmental stimuli and in signalling events [41,42,43].

A few concerns still exist about the reliability of these pharmacological approaches, especially in deciphering cAMP-dependent signalling mechanisms. Indeed, these methodologies do not consider the importance of cAMP physiological concentrations, which are well below the exogenously applied cAMP at micromolar levels. Hence, secondary effects cannot be excluded [16]. Moreover, the pharmacological approach cannot fully dissect cAMP-activated signalling pathways, as it does allow taking into account endogenous cAMP fluctuations.

More recently, a sophisticated molecular approach was successfully applied in animal systems to investigate simultaneous multiple signalling pathways: the engineering of a buffering molecule able to selectively bind one specific component of the investigated system, directly inside the cell [31,46]. It is the case of the first genetically encoded buffer for cAMP, called “cAMP-sponge”, based on the high-affinity cAMP binding portions of the regulatory subunits of human protein kinase A (PKA-RIβ) [31]. The PKA-RIβ C-terminus binds cAMP with high affinity, but it is unable to generate dimers or to bind the PKA catalytic inhibitory domain located at N-terminus [31,47]. The choice of the human PKA domains to buffer cAMP was well considered: the affinity had to be high enough to compete with endogenous effectors of the cAMP signal (Epac, PKA and CNGCs), but lower than the resting free levels of cAMP. Furthermore, the fragment was tagged with the fluorescent protein mCherry, which is spectrally compatible with FRET-based sensors for cAMP, allowing the simultaneous detection of both expressed buffer and cAMP, at a single cell level. The recombinant probe can also be specifically targeted to a specific subcellular compartment. Lefkimmiatis and co-workers generated both a non-targeted construct and a cytosolic cAMP-sponge construct, the latter bearing the N-terminal nuclear exclusion signal, highlighting the possibility of restricting the cAMP sponge expression to cell compartments by the addition of targeting motifs [31]. 

The cAMP-sponge was shown to bind specifically cAMP in vitro with sub micromolar affinity and it was insensitive to cyclic cGMP (3′,5′-cyclic guanosine monophosphate). It was validated at the single cell level, using a FRET-based imaging approach. The cAMP sponge was able to buffer agonist-induced cAMP signals and to block the downstream activation of PKA [31]. This molecular approach offered the opportunity to give a glance on cAMP functioning in living cells, providing information about endogenous cAMP changes.

The cAMP-sponge has been recently used as non-invasive tool in two plant model organisms, *Nicotiana tabacum* Bright Yellow-2 (BY-2) cells and *Arabidopsis thaliana* plants, to obtain a new portrayal of cAMP role in plants, through the in vivo depletion of cAMP in plant cells [32,33].

**Figure 1:**
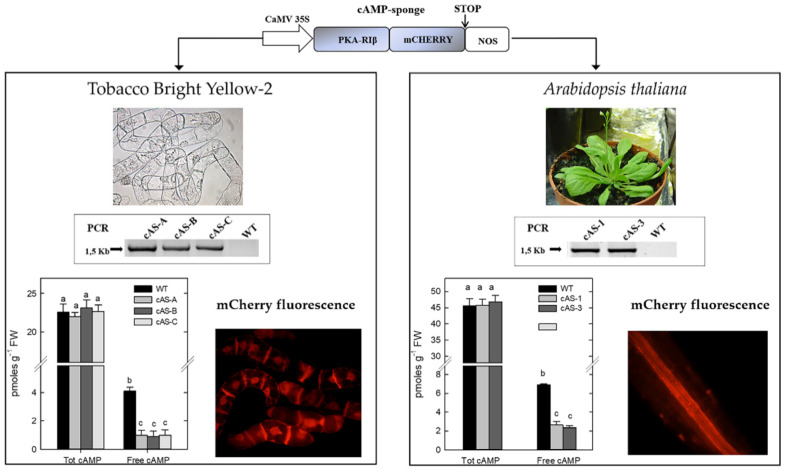
Schematic representation of the “cAMP sponge” overexpression in *Nicotiana tabacum* Bright yellow-2 (BY-2) cells and *Arabidopsis thaliana* plants. The cAMP-sponge construct used for tobacco BY-2 and Arabidopsis genetic transformations is reported on the top of the figure. The two following panels illustrate the characterisation of different transgenic lines (cAS lines) overexpressing the cAMP sponge in tobacco BY-2 cells (left) and in Arabidopsis plants (right). RT-PCR products show the integration of the transgene in the transformed cAS lines. Total and free cAMP content in wild type (WT) and cAS lines are reported in the histogram graphs. The presence of cAMP sponge protein is visualised by mCherry fluorescence. (Adapted from Sabetta et al. (2016) and Sabetta et al. (2019) [32,33]).

In both model systems, the cAMP-sponge was successfully expressed under the control of the 35S Cauliflower Mosaic Virus (CaMV) constitutive promoter. Three and two stable and independent transformed lines (cAS lines) of transgenic tobacco BY-2 cells and Arabidopsis plants, respectively, were obtained (Figure 1). The integration of the transgene in the nuclear genome and the in vivo presence of the cAMP-sponge protein were confirmed by the detection of the mCherry fluorescence. The transgenic lines showed the same total cAMP content of the wild type (WT) ones, likely because the cAMP bound to the cAMP-sponge was released during the procedure of total cAMP extraction. By contrast, the measurement of free cAMP content showed significant differences, with transgenic cAS lines displaying about half the free cAMP compared with WT lines (Figure 1).

The characterisation of the transgenic cAS lines of tobacco BY-2 cells showed that cAMP dampening inhibited cell growth and this was due to mitosis inhibition, rather than a decrease in cell viability. Moreover, transgenic cells showed enhanced antioxidant levels indicating that these cells sense cAMP deficiency as a stress condition [32]. On the other hand, cAS Arabidopsis transgenic lines did not exhibit any phenotype in physiological conditions, showing the same germination time, number, colour and size of rosette leaves, as well as time and height of inflorescence as WT lines. These observations supported a non-pleiotropic effect of cAMP-sponge and the specificity of this genetic approach [33]. A comprehensive proteomic analysis conducted on the transformed tobacco BY-2 cells in the exponential phase of growth highlighted that 29 and 65 proteins were over- and under-accumulated, respectively, compared to WT cells [32]. By contrast, the proteomic analysis on Arabidopsis leaves from six-week-old plants indicated that only four proteins were differentially accumulated in cAS plants compared with WT, and among these phospholipase C was heavily downregulated [33]. Despite the absence of phenotype at resting conditions, cAS Arabidopsis plants showed reduced resistance to the avirulent pathogen *Pseudomonas syringae* pv. tomato DC3000 carrying the avirulence gene AvrB (PstAvrB), confirming that cAMP is required for the correct immune response activation [33].

These findings demonstrate the potential of the cAMP-sponge tool to unravel cAMP roles and signalling mechanisms in plants.

## 3. cAMP in Plant Physiological Processes

**Figure 2:**
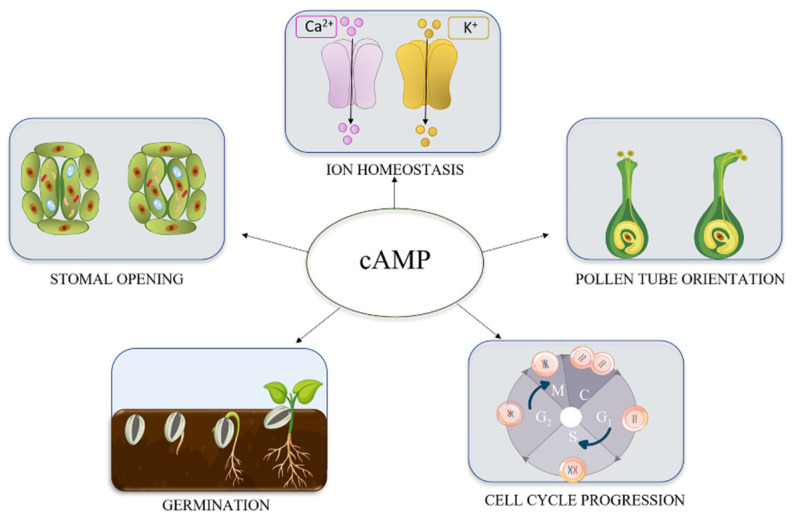
cAMP involvement in plant physiological processes. Literature data indicate a role for cAMP in ion homeostasis, mainly through the regulation of membrane-localised ion channels [29,34,35,36,40,48,49,50,51,52,53,54] and in stomatal opening, through Ca^2+^ and K^+^ flux regulation [39,40,52,53], cAMP was also shown to influence pollen tube orientation and growth, by the regulation of Ca^2+^ channels and choline acetyltransferase activity [14,54,55,56,57,58]. Seed germination [59,60,61,62,63] and cell cycle progression [32,33,37,38,64] are also regulated by cAMP. More details are provided in the text.

Although in plants the key actors of cAMP signal transduction are still not well defined, increasing evidence demonstrates that cAMP could affect several physiological processes (Figure 2).

Many papers highlight that cAMP is a potential regulator of ion homeostasis. The first substantial evidence for this cAMP function was obtained through the observation of whole-cell patch-clamp current in *Vicia faba* mesophyll after the application of 1 mM or higher concentrations of cAMP. Since this concentration is higher than the physiological cAMP concentrations, Li and co-workers supposed that plant cells could have high levels of PDE activity that hydrolyses the exogenous cAMP. They demonstrated that the application of micromolar cAMP concentration alongside the PDE inhibitor isobutyl-1-methylxanthine (IBMX) modulated an outward K^+^-current. Moreover, using inhibitors of animal PKA and the catalytic subunit of PKA, the authors obtained indirect evidence that the modulation of K^+^ channel activity could be mediated by a cAMP-regulated protein kinase [45]. Moreover, cAMP decreased cytosolic calcium in guard cell protoplasts, enhancing stomatal aperture in both light and darkness, in a protein kinase-dependent manner [39]. A successive work showed that cAMP participates in stomatal opening, antagonising the effect of abscisic acid (ABA) and Ca^2+^ on the inhibition of the inward K^+^-current [40].

A clear demonstration of changes in Ca^2+^ homeostasis and subsequent protoplast swelling in response to cAMP was shown in *Nicotiana plumbaginofolia*. It should be noted that the same effects were obtained with cGMP. Both cyclic mononucleotides induced a raise in cytosolic Ca^2+^, suggesting that the release of intracellular and extracellular Ca^2+^ stores acted as a signal at the crossroad of transduction pathways of these second messengers [48]. A direct effect of cAMP in regulating calcium conductance in leaf guard and mesophyll cells was shown in Arabidopsis [49]. By means of excised outside-out patches, Lemtiri-Chlieh and colleagues showed that the addition of permeable cAMP analogues stimulated a channel with fast gating kinetics. The results indicate that the increase of cytosolic Ca^2+^ was due to a plasma membrane-localised Ca^2+^ channel, suggesting the existence of functional CNGCs in these cells [49].

More recently, it was recognised that cAMP-dependent alteration of ion homeostasis could occur through the binding of CNGCs, which represent key sites where cyclic nucleotide interacts with ion signalling pathways [29,50]. For instance, the cAMP-activated inward of Ca^2+^ current through the plasma membrane is impaired in leaves of the CNGC2 loss of function mutant, *dnd1* [51]. CNGC2 and influx of apoplastic Ca^2+^ was also shown to be implicated in jasmonic acid-dependent rise in cytosolic cAMP, involved in the signalling of Arabidopsis guard cells [52,53].

A transient rise in cytosolic Ca^2+^ concentration after cAMP addition also occurred in pollen tube of *Agapanthus umbellatus* [54]. In the same species, cAMP was proposed as a signalling molecule involved in pollen tube reorientation. Growing pollen tubes showed an uniform cAMP concentration of 100–150 nM and changes in tube growth direction resulted from transient elevation in the apical region. The cAMP changes are due to PSiP, a putative AC cloned from *A. umbellatus* pollen. The antisense assays, achieved with oligos against this AC, caused the loss of pollen tube growth, suggesting that cAMP synthesis was a requirement for this event [14]. In *Pyrus pyrifolia*, through patch-clamp studies, it was shown that cAMP activated Ca^2+^ channel with a consequent increase in cytosolic Ca^2+^ of pollen tube protoplast. This event was specific for cAMP since cGMP failed to provoke the same effect. The cAMP-dependent opening of plasma membrane Ca^2+^ channels and the cytosolic Ca^2+^ increase affected pollen tube growth [55]. A different mechanism for cAMP control of pollen tube elongation was proposed in *Lilium longiflorum*. Application of exogenous cAMP at physiological concentration, as well as AC activators and PDE inhibitors, promoted the elongation of pollen tubes after self-incompatible pollination [56,57]. In addition, the content of endogenous cAMP in pistils after self-pollination was lower than that observed with cross-pollination and this difference reflected the different activities of AC and PDE [57]. Successively, it was demonstrated that cAMP stimulated the activity of choline acetyltransferase, which controls the synthesis of acetylcholine, a molecule that, together with other choline derivatives, promotes the elongation of *Lilium longiflorum* pollen tube. Thus, the low levels of cAMP and the subsequent low activity of choline acetyltransferase caused the self-incompatibility in *Lilium longiflorum* [58].

A pivotal role for cAMP in the control of cell cycle progression and cell division was also reported. In synchronised tobacco BY-2 cells, peaks of cAMP level were observed in S and G1 phases of cell cycle. The treatment with indomethacin, which is an inhibitor of prostaglandin-dependent adenylyl cyclase in animal cells, inhibited cAMP accumulation and mitosis [37]. The data on the expression of histone H4 and cyclin A, together with flow cytometric analyses, showed that indomethacin inhibits G1/S transition. [38]. However, the addition of exogenous cAMP failed to rescue indomethacin blocked cells, suggesting that indomethacin might affect other prostaglandin regulated activities [37]. The need to inhibit cAMP accumulation with methods independent of prostaglandin metabolism was overcome by using BY-2 cells overexpressing the cAMP sponge [32]. In vivo cAMP dampening in BY-2 cells caused a reduction in cell growth, mainly due to the mitosis inhibition, which occurred in parallel with a reduction in the cytoskeletal proteins, alpha- and beta-tubulin and actin depolymerisation factor, which are critical for cell division [65]. In parallel with cAMP deficiency, the expression of cell cycle genes was downregulated, suggesting that mitotic inhibition was due to a delay in cell cycle progression, which can occur at the G1/S and G2/M checkpoints. The delay of cell cycle progression was also supported by proteomic analysis [32]. The need of cAMP for a correct cell cycle progression and mitosis was also shown with pharmacological approaches in two-day-old seedling roots of *Raphanus sativus*. Domanska and colleagues suggested that different concentrations of cAMP are required for the start of DNA replication and mitosis and that cAMP can be involved in cell cycle transition during both replication and mitosis phases [64]. cAMP-dependent regulation of cell proliferation and differentiation was proposed for the formation of leguminous roots nodules. Plants with symbiotic nodules contained high levels of cAMP in the root nodules and cAMP contents increased during nodule development and decreased with nodule senescence [66,67].

Another possible role of cAMP in higher plants is the promotion of seed germination, suggested by the relationship observed between this second messenger and gibberellins (GA). The first evidence of this relationship was noticed in barley aleurone layers, where cAMP was shown to be able to substitute GA in the induction of α-amylase [63]. Early studies also showed that both cAMP and GA promoted germination of light-sensitive lettuce seeds and mannitol-treated weed seeds [60,61]. More recently, it was shown that cAMP acts downstream GA in the germination of the root parasitic plant *Orobanche minor* [62]. The *O. minor* seeds, prior to exposure to stimulants released from roots of host plants, need conditioning, which is a preincubation in a warm moist environment. Endogenous cAMP accumulated in the conditioned seeds. Moreover, exposure to light or supra-optimal temperature, throughout the conditioning period, led to cAMP decrease and low germination rates, which could be restored by GA treatments [62]. Similar results were also revealed during the seed germination of non-parasitic plant *Phacelia tanacetifolia* [63]. Under optimal light and temperature conditions, the seeds showed a transient cAMP accumulation before germination, which could be blocked by an inhibitor of GA biosynthesis. When the seeds were exposed to non-optimal conditions, inhibition of cAMP accumulation and germination occurred. Thus, cAMP could play a key role in favouring or blocking germination in response to environmental signals [63].

## 4. cAMP Involvement in Plant Response to Abiotic Stress

The establishing of plant responses to environmental stimuli requires the activation of multiple reactions at gene, transcript and protein level, interconnected by the action of signalling messengers [68]. In environmentally stressed plants, cellular metabolism faces a remarkable rearrangement allowing stress acclimation. Early alarm stages of plant abiotic stress response include the onset of oxidative stress and the induction of stress-responsive signalling pathways. Following the acclimation phase, with the biosynthesis of stress-protective compounds, cells encounter new recovering homeostasis, at the expense of cellular energy [68,69,70]. In this scenario, cAMP may act as stress sensors and/or modulator of cellular metabolism, mainly, but not only, through its influence on ion channels and the resulting regulation of ion fluxes [16] (Table 1).

In Arabidopsis, the improvement of plant salinity tolerance involves cAMP, which causes the deactivation of voltage-independent non-selective channels, limiting Na^+^ influx [71]. In wheat, tolerance to aluminium requires cAMP-dependent outward-rectifying K^+^ current, which permits malate outflow that chelates this toxic metal [73].

The important link between K^+^ flux and cAMP production was further defined in *Arabidopsis thaliana* by the isolation and characterisation of two K^+^-uptake permeases, AtKUP5 and AtKUP7. Both the K^+^-uptake permeases have a dual function, harbouring also a functional AC catalytic domain [20]. AtKUP7 is a K^+^ transporter in roots, functionally active under K^+^-limited conditions [77,78]. In addition, AtKUP7 was defined as a proton-coupled carrier with AC function, but it is still unclear if cAMP production is dependent on K^+^ fluxes and/or if cAMP can modulate K^+^ fluxes [20]. AtKUP5 causes a K^+^ flux-dependent cAMP accumulation in the cytosol, which can in turn activate downstream components essential for K^+^ homeostasis, including CNGCs [21].

cAMP involvement in abiotic stress response often goes through the regulation of CNGCs [29]. Remarkably, these ion channels, having overlapped binding domains for cyclic nucleotides and calmodulin, favour the crosstalk between the signalling of these second messengers [79,80]. Functional characterisation of Arabidopsis CNGC2 shows that cAMP activation of AtCNGC2 currents could be reversed by calmodulin, suggesting that the physical interaction of Ca^2+^ and calmodulin with CNGCs stops cyclic nucleotide activation of the channels. Therefore, the cytosolic cAMP, Ca^2+^ and calmodulin can operate in an integrated way to gate currents through CNGCs. [81].

CNGCs allow the influx of K^+^, Na^+^ and Ca^2+^ into the cell, with different selectivities; hence, they work downstream the environmental stimuli perception to mediate plant tolerance to drought, salinity and extreme temperature, which affect ionic and osmotic cellular homeostasis [29]. AtCNGC2 was shown to partially complement the yeast mutant at low K^+^ concentration only in the presence of membrane-permeable cAMP [82]. AtCNGC10, AtCNGC19 and AtCNGC20 were shown to be involved in plant tolerance to salt stress [83,84]. The antisense lines of AtCNGC10 showed altered K^+^ and Na^+^ levels in shoots and were less tolerant to salt stress [83]. AtCNGC19 and AtCNGC20, participating in the re-allocation of Na^+^ in the plants, might permit their survival to high salt levels [84].

Arabidopsis CNGC16 was shown to confer thermotolerance to germinating pollen, linking cyclic nucleotide signalling to heat stress response. In the *cngc16* mutants, the reduced transmission of pollen at high temperature was linked to a weakened expression of crucial stress-responsive genes. [85]. The role of CNGCs in plant thermotolerance was also validated in the vegetative tissue of plants. Mutants in CNGC2 showed hypersensitive heat-responsive Ca^2+^ influx, which conferred acquired thermotolerance at milder heat stress than in wild-type plants [86]. Mutation in Arabidopsis CNGC6 led to impaired heat stress response, which suggests its involvement in the acquisition of thermotolerance [74]. In addition, in Arabidopsis, it was shown that a heat shock caused an increase in intracellular cAMP levels, which, in turn, stimulating CNGC6, triggered a cytosolic Ca^2+^ influx. Furthermore, the treatment with an exogenous cAMP analogue induced the expression of some heat shock proteins, indicating the contribution of this second messenger in plant heat stress response [74].

Proteomic studies also supported a role of cAMP in controlling plant response to temperature, as well as to light. Thomas, Alqurashi, and their colleagues, suggested that cAMP participates as signalling molecule to the photosynthetic process of acclimation. [41,42]. The analyses revealed that, after cAMP treatment, the most enriched proteins belonged to the GO categories “Response to stress”, “Response to abiotic stimulus”, “Response to salt” and “Response to cold”. Moreover, there was an enrichment of the category “Photosynthesis and light reaction processes” in both up- and downregulated cAMP responsive genes [41]. cAMP involvement in photosynthetic pathways was also described by Donaldoson and colleagues [43], who reported the interaction between cAMP and enzymes involved in Calvin cycle and photorespiration pathway. This is of interest since in *Nicotiana tabacum*, through a quantitative method based on mass spectrometric analysis, AC activity was observed in chloroplasts [87]. Moreover, in oat seedlings, it was shown that light influenced cAMP accumulation, pointing out that cAMP could take part in the phytochrome signalling pathway [88].

A role for cAMP in plant response to drought was also proposed in wheat. Indeed, the exogenous application of both cAMP and ABA promoted the synthesis of polypeptides whose accumulation is stimulated by dehydration, suggesting that cAMP signalling is possibly involved in the effect of ABA on protein synthesis during drought [75].

cAMP was shown to be involved in response to wounding in *Hippeastrum x hybridum*. In this plant, the transcriptional activity of the HpAC1 gene, which encodes a functional AC, as well as the level of cAMP, showed two peaks in response to mechanical damage. The authors proposed that the first rapid induction of HpAC1, and the concomitant transient changes in cAMP, might function as an “alarm” that alerts plant cells against the damage. The later increase in HpAC1 expression and cAMP accumulation might be linked to the induction of systemic responses and, in particular, to the induction of phenylalanine ammonia lyase (PAL) involved in the production of phytoalexins, which protect damaged tissue against potential pathogen attacks [76]. Together with PAL induction, cAMP was shown to be involved in the stimulation of the expression of 4-coumarate:coenzyme A ligase and chalcone synthase, enzymes of the phenylpropanoid pathway, which participates to plant response to a multiplicity of environmental stimuli, including nutrient depletion, UV irradiation, extreme temperatures and heavy metal toxicity [89].

Oxidative stress is a common feature associated with various abiotic stress factors, and reactive oxygen species (ROS) have an important biological role in sensing and activating acclimation mechanisms [68,90,91]. The superoxide-generating NADPH oxidase integrates Ca^2+^ and ROS signalling, which in turn may be connected to cyclic nucleotides through CNGCs [92]. Each messenger mutually enhances the induction of the other during abiotic stress conditions, resulting in the propagation of ROS and Ca^2+^ waves across the plasma membrane to establish the proper acclimation response, to which cAMP may directly or indirectly participate [93,94].

A correlation among cAMP, ROS and ion homeostasis was demonstrated in plant response to salt stress [72]. Several studies indicated that, in roots under salt stress, ROS accumulation could be due to the disturbance of mitochondrial function, as well as to activation of NADPH oxidases [95,96]. Furthermore, under salt stress, Na^+^-influx into the cell causes a significant loss of cytosolic K^+^, which can be responsible for important metabolic alterations [97,98]. The treatment of Arabidopsis roots with H_2_O_2_ induced a rapid Ca^2+^-influx and K^+^-efflux, which were reduced by pre-treatment with cAMP. Moreover, coherently with the accumulation of H_2_O_2_ level in salt-stressed roots [95,96], pre-treatment with cAMP decreased salt-dependent K^+^-efflux [94]. Ordonez and colleagues proposed that CNGCs, proved to be involved in plant responses to salt stress [84], could be in part responsible of the H_2_O_2_-dependent K^+^- efflux, which was reduced by cyclic nucleotides [72].

## 5. Role of cAMP in Plant Innate Immunity

Plants are continuously exposed to a variety of invading microorganisms, including viruses, bacteria and fungi. Although plants are lacking mobile sentinel cells, distinctive of the animal immune systems, they can perceive and keep away pathogens, through a two-layer innate immune system [99]. In the first layer of defence, called pattern-triggered immunity (PTI), membrane pattern recognition receptors (PRRs) recognise pathogen/microbe-associated molecular patterns (PAMPs/MAMPs) or endogenous damage-associated molecular patterns (DAMPs) [100,101]. This recognition initiates a series of defence responses, including ROS production, Ca^2+^ influx and activation of kinases as Ca^2+^-dependent protein kinases and mitogen-activated protein kinase, leading to the upregulation of defence genes [101,102]. However, pathogens can secrete into plant cells effectors, namely virulence factors encoded by avirulence (avr) genes, which can suppress PTI. The effector recognition by intracellular receptors encoded by resistance genes activates the second layer of defence, the effector-triggered immunity (ETI). Defence responses of ETI are typically stronger than PTI and often culminate with the hypersensitive response (HR), a form of programmed cell death, occurring at the infection site with the aim to narrow pathogen infection [99,103]. An increase in the antimicrobial phytoalexins, as well as in salicylic acid (SA) and pathogenesis-related (PR) proteins, occurs locally in the site of infection, and systemically in uninfected tissues [104].

**Figure 3:**
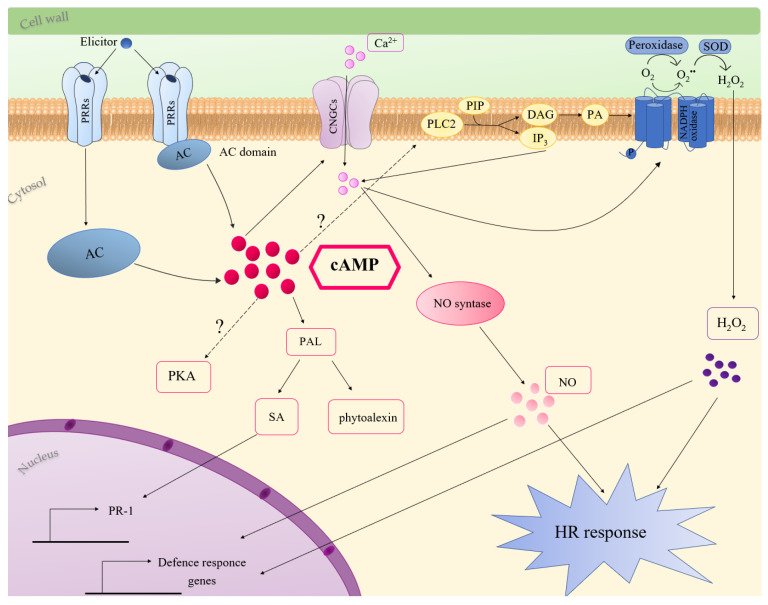
Molecular mechanisms of cAMP involvement in plant innate immunity. Elicitor recognition elevates cytosolic cAMP, which can activate CNGCs or PLC2, inducing Ca^2+^ accumulation and oxidative burst, through the activation of NADPH oxidase. cAMP-dependent oxidative burst can also be due to apoplastic peroxidases. Ca^2+^ stimulates NO production, which, together with ROS, induces defence response and HR. cAMP accumulation also activates PAL expression and production of SA and phytoalexins. More details are provided in the text. Question marks indicate pathways not completely characterised. Abbreviations: AC, adenylate cyclase; cAMP, 3′,5′-cyclic adenosine monophosphate; CNGCs, cyclic nucleotides-gated channels; DAG, diacylglycerol; HR, hypersensitive response; IP_3_, inositol triphosphate; NO, nitric oxide; PA, phosphatidic acid; PAL, phenylalanine ammonia lyase; PIP, monophosphatidylinosotol; PKA, protein kinase A, PLC_2_, phospholipase C_2_; PR-1, pathogenesis-related genes; PRRs, pattern recognition receptors; SA, salicylic acid; SOD, superoxide dismutase.

Several studies indicated the involvement of cAMP in plant immune response [33,105,106,107,108,109,110]. Considering all the literature data until now reported, possible cAMP-mediated mechanisms activated during plant-immunity are discussed (Figure 3).

Initially, a role for cAMP in the biosynthesis of phytoalexins was proposed. In carrot cell culture, the addition of the permeable dibutyryl cAMP, or forskolin and cholera toxin, activators of adenylate cyclase and G proteins, respectively, induced the biosynthesis of the antifungal phytoalexin 6-methoxymellein. Interestingly, the cAMP-dependent production of this phytoalexin was inhibited by Ca^2+^ channel blockers, as well as by inhibitors of calmodulin-dependent processes, suggesting that the increase in cAMP content in carrot cells induces Ca^2+^ influx across the plasma membrane [44,105]. In *Cupressus lusitanica* cell cultures, cAMP is involved in elicitor-induced production of the phytoalexin, β-thujaplicin. The authors suggested that cAMP-dependent β-thujaplicin accumulation involves Ca^2+^ and K^+^ fluxes since it was inhibited by K^+^ and Ca^2+^ channel blockers. This study also indicated a contribution of protein kinase cascades in cAMP signalling processes leading to β-thujaplicin accumulation [107]. The cAMP-dependent production of phytoalexins was also shown in *Medicago sativa*. In this case, the treatment with an elicitor of the phytopathogenic fungus, *Verticillium alboatrum*, caused a dose-dependent increase in the activity of AC and in intracellular cAMP content. Moreover, the treatment of Medicago cells with cAMP enhanced PAL activity and the synthesis of the phytoalexin medicarpin [106]. Consistently, in Arabidopsis, the treatment of seedlings with the permeable cAMP analogue 8-Br-cAMP increased, up to 40-fold and 2-fold, respectively, the expression of PAL2 and PAL1 [87]. PAL, the expression of which increased in response to diverse pathogens and elicitors, also plays a key role in SA synthesis [111,112,113] (Figure 3). Remarkably, cAMP elevation in Arabidopsis increased the endogenous SA level in response against Verticillium secreted toxins. The treatment of Arabidopsis with an AC inhibitor strongly reduced SA accumulation and PR-1 expression caused by Verticillium toxins. Both 8-Br-cAMP and SA enhanced resistance of Arabidopsis to the toxins, but cAMP acts upstream SA, since it was not able to potentiate the resistance of Arabidopsis plants deficient in SA [108]. In line with a role for cAMP in SA-dependent defence responses, the upregulation of PR-1 gene expression, occurring in response to an avirulent strain of *Pseudomonas syringae*, was decreased in cAS plants with low cAMP levels [33].

During plant immune responses, an oxidative burst arises in two phases, the first occurring within few minutes after pathogen perception and the second occurring later and with a higher amplitude [114]. ROS play several roles in response to pathogens, such as the reinforcement of cell wall, the activation MAP kinase pathways, the induction of HR and the triggering of systemic responses [115,116]. Two main mechanisms including NADPH oxidases and peroxidases have been proposed for ROS generation in response to pathogens [116,117]. Many literature data suggest an involvement of cAMP in pathogen/elicitor induced oxidative burst (Figure 3). In French bean cell culture, cAMP level increased upon the addition of an elicitor of the fungus *Colletotrichum lindemuthianum* and cAMP itself induced ROS accumulation. The cAMP-mediated apoplastic oxidative burst was increased by cholera toxin and inhibited by Ca^2+^ channel blockers. Bindschedler and co-workers suggested that G proteins and cAMP are involved in extracellular alkalisation and Ca^2+^ influx, essential for the pH-dependent apoplastic peroxidases, which mediate the oxidative burst [118]. Likewise, the treatment of *Arabidopsis thaliana* cells with forskolin enhanced the oxidative burst occurring in response to an elicitor from *Fusarium oxysporum* [119]. ROS generation induced by the PAMP lipopolysaccharide in Arabidopsis was prevented by the addition of an AC inhibitor [109]. Similarly, cAMP dampening in Arabidopsis cAS plants caused a delay in H_2_O_2_ increase at the early stage of response to an avirulent strain of *Pseudomonas syringae* [33].

Genetic evidence supports a role for CNGCs in pathogen-induced HR and disease resistance (Figure 3). In Arabidopsis, the mutation in DND1 (defence-no-death), which encodes AtCNGC2, failed to induce HR in response to an avirulent strain of *P. syringae*. Moreover, *dnd1* mutants showed constitutive systemic resistance and elevated levels of SA [120]. HLM1, encoding AtCNGC4, which works as a K^+^- and Na^+^-permeable channel activated by cGMP or cAMP, was upregulated in response to pathogen infection. *hlm1* mutant plants showed a lesion-mimic phenotype and an altered HR in response to avirulent *P. syringae* pv tomato (Pst) strains harbouring the avrRps4 or avrRpm1 genes [121]. In Arabidopsis, cAMP-activated AtCNGC11 and AtCNGC12 are positive mediators of resistance against the avirulent *Hyaloperonospora parasitica*. In the *cpr22* (constitutive expresser of PR genes22) mutant, a 3-kb deletion that fuses AtCNGC11 and AtCNGC12, generates the chimeric gene ATCNGC11/12, which confers the constitutive activation of defence responses [122].

An increase in cytosolic Ca^2+^, due to influx across the plasma membrane or to efflux from intracellular stores, represents a primary event in plant immune signalling [123,124,125,126]. Interestingly, in *dnd1* mutant cells, the deficiency of cAMP-activated inward Ca^2+^ influx is associated with reduced production of nitric oxide (NO) [51], which was defined as the concertmaster in the HR and defence-gene activation [127,128]. *dnd1* mutants showed a weakened HR, and the addition of exogenous NO complements this phenotype [51]. Application of pathogens or PAMPS elevated cytosolic cAMP and the addition of exogenous cAMP led to Ca^2+^ elevation, NO generation and defence response in the absence of the non-self pathogen signal. Inoculation of *dnd1* plants with Pst containing the avrRpm1 or avrRpt2 genes led to a reduction in Ca^2+^ influx and to an impairment in immune response [51,109]. The weakening of pathogen-associated cytosolic Ca^2+^ influx also occurred by blocking cAMP synthesis in plants exposed to the pathogen, with a corresponding impairment in HR. On the contrary, co-infiltration with IBMX along with avirulent pathogens enhanced plant immune response, increasing HR. Thus, it was suggested that elevation of cytosolic cAMP, acting upstream from Ca^2+^, is a key signal in the transduction of pathogen perception and in the downstream signalling cascade of defence responses [109]. Furthermore, the cAMP dampening, occurring in Arabidopsis cAS plants, delayed cytosolic Ca^2+^ elevation and reduced HR in response to PstAvrB. Sabetta and co-workers suggested that the delay in Ca^2+^ elevation could be due to a failure in the activation of CNGCs, but also to the down-accumulation of phospholipase C2 (PLC2) occurring in cAS plants [33] (Figure 3). Consistently, it is known that cytosolic Ca^2+^ accumulation in response to numerous elicitors of plant defence involves phosphatidylinositol-specific PLCs [125]. Moreover, since it was reported that PLCs significantly contribute to pathogen/elicitor induced oxidative burst [129,130,131], the low level of PLC2 in cAS plants could also contribute to the delayed H_2_O_2_ increase in the first phase of PstAvrB infection [33]. The low availability of cAMP, and the subsequent delay in Ca^2+^ influx, could be responsible for an incorrect temporal modulation of the AtSR1 [33], a Ca^2+^-dependent calmodulin binding transcription factor, repressing the expression of target genes [131,132,133]. Consequently, some defence proteins, such as HSP90, CRK14 and DJ1E [134,135,136,137], were not accumulated in cAS cells after pathogen infection, weakening defence response [33].

The involvement of cAMP in plant immunity was supported by the isolation of ACs involved in plant response to pathogens. The silencing of NbAC, a gene encoding an AC in *Nicotiana benthamiana*, suppresses the necrotic lesions induced by tabtoxinine-β-lactam, a non-specific bacterial toxin, produced by *P. syringae* pv. Tabaci [138]. The expression of HpAC1, a gene encoding an AC from *Hippeastrum x hybridum*, and the levels of cAMP, increased in response to *Phoma narcissi* infection [76]. Recently, a leucine-rich repeat protein, AtLRRAC1, harbouring multiple catalytically active AC centres, was identified in Arabidopsis. AtLRRAC1 was able to complement AC-deficient *Escherichia coli* and to generate cAMP in vitro [18,22]. Interestingly, *atlrrac1* mutants showed compromised immune responses to biotrophic fungi and hemibiotrophic bacteria. The expression of early-induced immune-related genes after elicitation with the PAMP flg22 was strongly inhibited in *atlrrac1* plants, suggesting an involvement of AtLRRAC1 in PTI [22].

## 6. Conclusions

cAMP is the object of intense scientific interest, both in animal systems, where much more progress was achieved in defining its role, and in plants, becoming lately the centre of a bustling research. cAMP is nowadays recognised as a relevant signalling molecule in plant development as well as in responses to environmental stimuli, of both biotic and abiotic nature. As cAMP-signalling networks and their spatial and temporal regulation are extremely complex, future research must deal with the nature of cAMP signals in terms of strength, duration and frequency, considering also the crosstalk between this second messenger and other intracellular regulators [139]. Since the existence of cAMP-regulated processes in plants and the first evidence of compartmentalised cAMP signals in animals, the need for reliable cAMP detection methods able to reveal cAMP waves in living systems arose. Recent advances in modern biotechnologies and synthetic biology, alongside newly developed detection methods and instrumentations, offer a wide range of possibilities to unravel cAMP role in living cells.

The cAMP-sponge represents a cutting-edge genetically encoded tool, used to exploit cAMP fluctuations for the first time in living plant organisms and specific cell compartments. It overcomes major concerns on biochemical assays and pharmacological studies performed so far in plants [31,32,33]. Other developed genetically-encoded tools employed in bacteria and isolated plant cells are the promoter reporter systems, based on the plant protein Oligopeptide TransporterX promoter, which measure alterations in downstream gene expression following changes in intracellular levels of cyclic nucleotides. Unfortunately, this system cannot discriminate between cGMP and cAMP [140].

Taking advantage of the progress reached in animal systems, many other strategies and their combination may help in elucidating cAMP signalling in plant systems. Indeed, optogenetic approaches and genetically encoded fluorescent biosensors are effectively used to monitor and modulate cAMP levels [141,142]. Photoactivated ACs and light-regulated PDEs, or even their association, are successfully used in animal cells [143,144]. The generation of stable plant lines, expressing the combination of optimised sensors for cAMP and concomitant or downstream messengers, may provide a comprehensive view of the signalling event investigated.

Another important requirement is a clear identification and functional characterisation of cAMP-binding proteins involved in the signalling of this second messenger. Nowadays, many lines of evidence indicate that, in plants, the conversion of cAMP into Ca^2+^ signals via CNGCs is the main signalling mechanism of this cyclic nucleotide. However, although indications for bona fide PKA are lacking, its presence in plants cannot be excluded. New bioinformatics algorithms and molecular tools may provide opportunities to extend the presently scarce knowledge of cAMP-dependent protein kinases [16,23]. Moreover, studies on cAMP-dependent changes in transcriptomes, proteomes and phosphoproteomes, as well as metabolomes, will improve the understanding of cAMP involvement in plant physiological processes, along with acclimation to adverse environmental conditions.

## Figures and Tables

**Table 1:** Proposed role of cAMP in the acquisition of stress tolerance.

Stress	Mechanisms	Molecular Players	References
Salinity	Limitation of Na^+^ influx	VICs; CNGCs	[71,72]
Aluminium	K^+^ current permitting malate outflux	Cation channels	[73]
K^+^ deficiency	K^+^ homeostasis regulation	AtKUP5; AtKUP7; CNGCs.	[20,21]
Heat	Ca^2+^ influx and HSPs expression	CNGCs; HSPs.	[74]
Drought	Synthesis of protective polypeptides	ABA signalling	[75]
Wounding	Regulations of the phenylpropanoid pathway	PAL; 4CL; CHS.	[76]
ROS	Reduction of Ca^2+^ influx and K^+^ efflux	CNGCs	[72]

Abbreviations: ABA, abscisic acid; CHS, chalcone synthase; 4CL, 4-coumarate:coenzyme A ligase; CNGCs, cyclic nucleotide gated channels; HSPs, heat shock proteins; PAL, phenylalanine ammonia lyase; VICs, voltage-independent non-selective channels.
